# Cariprazine treatment in patients with psychosis related to Parkinson’s Disease: a case report.

**DOI:** 10.1192/j.eurpsy.2023.2279

**Published:** 2023-07-19

**Authors:** M. Martín De Argila Lorente, G. Ruiz Manrique De Lara, M. González Guembe

**Affiliations:** Hospital Dr. Rodríguez Lafora, Madrid, Spain

## Abstract

**Introduction:**

Because of their mechanism of action, treatments used in Parkinson’s disease can lead to the development of non-motor symptoms, including neuropsychiatric manifestations such as psychotic symptoms and mood disturbances such as depression. Cariprazine is a new atypical antipsychotic, D3 receptor antagonist, with lower affinity for the 5HT2A receptor and higher affinity for alpha 1B adrenergic receptors than any other antipsychotic, which has been shown in preclinical studies that play an important role in ameliorating levodopa-induced psychotic symptoms in patients with Parkinson’s disease.

**Objectives:**

A case of a patient with psychosis related to Parkinson’s Disease and depressive symptoms is presented followed by a theoretical review on the topic.

**Methods:**

A case is presented with a bibliographic review.

**Results:**

A 66-year-old male was hospitalized in the psychiatric unit of short hospitalization with psychotic symptomatology and presence of depressive symptoms.

The patient presented in his medical history two previous hospitalizations with depressive clinic with psychotic symptomatology in 2018 and February 2022, the last one after been diagnosed with Parkinson’s disease in January 2022. The introduction of Parkinson’s Disease treatment with levodopa/benserazide caused a secondary worsening of his psychotic symptomatology. In addition to this, the patient was being treated with quetiapine, desvenlafaxine and clonazepam.

During the hospitalization, the dose of desvenlafaxine was increased and quetiapine treatment was replaced by cariprazine because of no clear improvement in symptomatology. After 24 hours, the patient showed a clear clinical improvement: better mood, delirious ideas decreased as well as the hearing of voices. No adverse events related with this medication were observed. After 20 days of hospitalization, the patient was discharged with favorable evolution.

**Conclusions:**

Although most of the studies available so far propose quetiapine as the antipsychotic treatment of choice for levodopa-induced psychosis in patients with Parkinson’s disease, the case report presented strengthens the recent data described in the literature on cariprazine in the treatment of psychosis related to Parkinson’s Disease. However, additional long-term studies including a larger number of patients with long-term follow-up will be necessary to confirm the efficacy of this drug in this type of patients with this pathology.

**Disclosure of Interest:**

None Declared

